# On the Yellow Fever of Fernando Po

**Published:** 1831-01-01

**Authors:** 


### On the Origin, Progress, and Termination of a malignant Yellow Fever, which lately affected the Crew of His Majesty's Ship Sybille on the Coast of Africa, &c. By *Robert M'Kinnal*, M.D. Surgeon R.N.

[Addressed to the Naval Medical Commissioners.]

This document, containing much valuable matter, having been liberally confided to our inspection by the Medical Commissioners of His Majesty's Navy, we shall endeavour to give a concise analysis of its contents in this article, in the same manner as we would of a printed work.

It appears that the crew of the Sybille had enjoyed uninterrupted health on the coast of Africa. They had been cruizing in the bights of Benin and Biafra, from December, 1828, till the 21st of June, 1829, when they arrived at Fernando Po. Here they found the Eden, which ship had lost her captain, surgeon, assistant-surgeon, and upwards of 50 men, by yellow fever at Sierra Leone, and on her passage to Fernando Po. The disease was still continuing its ravages in the ship at Clarence Cove. The Hecla had also suffered, as well as the Champion, lately arrived from England. The colonial surgeon and his assistant, who had come from England in the Champion, (which ship had accompanied the Eden from Sierra Leone) died soon after landing at Fernando Po. Yet the colony of Sierra Leone was reported healthy at the time the Champion sailed. All communication between the Sybille and the shore or the Eden, was prohibited during the stay of the Sybille, excepting the purser and one or two superior officers. On the 22d of June, however, they received on board a serjeant of the Royal Marine Artillery, and seven marines from the Eden, who came from England in the Champion; and on the following day they received from the establishment at Fernando Po, Charles Hall, a marine, who had also arrived in the Champion. On the 23d of June, John Mecking, received from the Eden the preceding day, became affected with fever, and was quickly sent ashore. On the same evening the Sybille sailed from Fernando Po, and, on the same day, the serjeant above alluded to was put on the sick list for rheumatism. To him and to the seven marines, ℥j. of cinchona, with a gill of wine, was administered twice daily for four days, and afterwards once a day for a few days longer. On the 26th of June, William Love, a boy, was seized with fever, though he had had no communication with the shore or with Mecking. The case was mild. On the 2d of July, at Prince's Island, Charles Hall, received from Fernando Po, was seized, and from this period the disease continued to arise in different parts of the ship, and while at sea. It soon assumed the most malignant character, and, in its progress, attacked individuals of every class, age, temperament, and colour. The negroes were affected with it in comparatively small numbers, and in a mild degree. The marines were the greatest sufferers. On the most minute investigation, the surgeon was unable to trace the disease, either from man to man or from mess to mess; and those who visited or attended the sick were not more affected than those who kept themselves most aloof from fever cases. The usual attendant on the sick berth, and the surgeon himself, escaped the fever, though both unprotected by any previous attack. The sail maker, who sewed up the dead bodies in their hammocks had a slight attack, though he had formerly had the yellow fever at Jamaica, while the boy who assisted him escaped, though equally exposed, and unprotected by any former attack. Every attention was paid to cleanliness and ventilation —and the dead bodies were speedily committed to the deep, together with their bedding and clothes.

The weather, during the prevalence of the fever, was thick and hazy. There were heavy dews by night, but very little rain. The thermometer ranged from 78 to 80 by day, and fell a few degrees at night. The last case of fever occurred on the 27th of August, on the passage to St. Helena, and the following day, while passing to windward of the Island of St. Thomas, the weather suddenly changed to a dry, pure, and clear atmosphere, and every one on board experienced a mental, as well as corporeal exhilaration, the thermometer still standing at the same range.

Two cases of fever occurred in June—twenty in July—and forty-seven in August—total 69 cases, of which number 22 died—or nearly 1 in 3. The disease was evidently yellow fever, in its highest degree of intensity. With two exceptions it was of the continued kind—the stage of excitement short, terminating, in the worst cases, in exhaustion and death on the third, fourth, fifth, or sixth day—but most frequently on the fifth. Death was preceded, in a great number of cases, by black vomit, often accompanied by a dingy or livid hue of the countenance. A peculiar, shrunk, corrugated and livid appearance of the extremities of the fingers, was not uncommon. Some died in convulsions, and others in a comatose state, without black vomit. A few went off with a dark-coloured, watery purging, often accompanied by syncope at stool and great exhaustion. Yellowness of the eyes and skin was very common before or after death. It varied from a pale lemon colour to a dark orange hue. An officer died, on the eleventh day, of relapse, though he had had the yellow fever in the West Indies. Some patients had hæmorrhages and ecchymoses. One man bubo in the groin, after the cessation of the fever.

It is worthy of remark, that of the eight marines received from Fernando Po, who took bark for eight days, two only became affected with fever. One went through all the usual symptoms of the yellow fever—recovered—and, when nearly fit for duty, had a relapse, the fever assuming the intermittent form, and readily yielding to quinine. The other marine was seized nearly a month after joining the ship, though unprotected by any former attack.

"The sudden cessation of new attacks (says Dr. M'Kinnal) of fever on the 28th August, when 40 men were on the list, and when the power of contagion, (if it existed) must have been at its height, seems to prove that atmospherical changes had great influence in the production, as well as in the extinction of the disease."

On the 12th September the ship arrived at St. Helena, without a man on the sick-list, being the seventeenth day from the date of the last seizure, and the third day from the last death by fever. After a couple of days' quarantine, the officers and men of the Sybille went on shore and mixed with the inhabitants of St. Helena, from the above date till the 25th of October, without any accident resulting from this unrestricted intercourse. On the 25th October the Sybille sailed for the Coast of Africa, all the crew in good health—touched at Ascension,—and anchored at Fernando Po on the 19th November. She remained only two days there, and none went on shore except Commodore Collier. The garrison was healthy. The Sybille touched at Prince's Island to take in some wood and water, occupying a couple of days, and then sailed on a cruize. On the 5th and 6th of December she was at anchor off Wydah, where bullocks were procured—and then she proceeded on her cruize, at a great distance from land. On the 3d January she anchored at Prince's Island, and joined the squadron all well. Here the Black Joke, tender to the Sybille, arrived from Sierra Leone, where she had been very sickly, and lost twenty-three men. The disease commenced like yellow fever, but afterwards assumed the form of the common remittent fever, affecting chiefly those men who had been in prizes, or who had volunteered from timber-ships in the river, or from Free Town, to serve in the Black Joke. All, however, were convalescent or entirely recovered, when this vessel arrived at Prince's Island. When the Sybille was weighing anchor, on the 7th January, a boy came on board from his ship the Tyne, who laboured under common tertian, which was readily cured. He stated that the Tyne was healthy. On the 13th January, yellow fever again broke out in the Sybille, while cruising off Cape Formosa. Dr. M'Kechnie, an assistant surgeon, who had lately come from England, and who had been for a few minutes in the Black Joke, was the first to take the fever. The disease soon increased in the ship, and early in February it became very alarming, producing the most dreadful havoc, "affecting, as formerly, individuals of every habit, temperament, age, and colour." The weather had been very hot and sultry, and the tornados commenced sooner than usual. The number of cases, during this visitation, amounted to 87, of which 26 died with the usual symptoms of the most malignant yellow fever.

Afterwards in St. Helena Roads, on the 22d March, the fever again broke out, and twenty-two cases occurred, of which six died. She sailed from St. Helena on a cruise, and during that time the Negroes on board were employed to give the hold and wings, &c. a thorough clearing out, succeeded by ventilation and all kinds of purification. After this there does not appear to have been any cases of fever in the Sybille.

"Before I conclude, I beg leave to observe that I am now of opinion, that the yellow fever in the Sybille, on both occasions, arose principally from noxious emanations from the interior of the ship herself, caused probably by the decomposition of the wood from the long-continued action of heat and moisture, and aided, perhaps, by an accumulation of different substances, which, after a certain time, must take place in a greater or less degree, particularly under the timber boards and lining of the ship, in all vessels whatever. It is probable also that the difference of health with respect to yellow fever, in different ships in tropical climates, placed apparently under similar circumstances in other respects, depends on the kind, age, and quality of the wood of which the vessel has been built, or with which she has been repaired. Atmospherical causes contribute also, no doubt, to the production of the disease, not only by favouring decomposition and the formation of noxious effluvia, but also by rendering the body more susceptible of their influence, or by acting as an exciting cause.

This opinion of the principal source of the disease in the Sybille is founded chiefly on the following considerations.

1mo. That there is not on record, as far as I know, an instance of yellow fever originating at sea, possessing great malignancy of character, and affecting a great number of men without communication with an unhealthy ship, (in which case the disease is usually, if not always confined to those who have visited such a vessel,) or exposure to contagion, or to noxious emanations from the shore in an unhealthy situation where the disease is often found to prevail, or from the ship herself.

2ndo. The immunity from fever of several other ships on the African station, though cruising nearly in the same situation as the Sybille, and exposed to the same, or similar weather.

3tio. Because it is impossible to ascribe it to an accumulation of filth in the holds or store-rooms, in a ship so clean, so well ventilated and regulated as the Sybille has been since she was last commissioned.

4to. The report that the Sybille has been an unhealthy ship for many years, both in the West Indies and in the Mediterranean.

5to. My firm conviction, from the facts above stated and others that might be added, that the disease was not contagious.

And lastly, because all the facts that I have stated, and particularly the occurrences at St. Helena, prove that the disease had, notwithstanding, a local origin."

We regret exceedingly that Dr. M'Kinnal has almost entirely blinked the important question respecting *second attacks*. During three visitations of the fever in one frigate, whose ship's company could not have exceeded 300 men at the utmost, 178 underwent fever; and yet the medical officer does not authenticate a single instance, as far as we can see, of a second attack. On the other hand, he frequently alludes to the immunity or protection of previous seizures, which leads us indirectly to the assumption that no secondary seizure occurred. We do not make this remark with any intention of inferring that the "Bulam fever"—the "nova pestis"—the "Gibraltar fever," was a specific disease, like small-pox or measles, affecting the constitution (generally speaking) but once. We state the fact fairly, in order to give the advocates of contagion every advantage that this fatal scourge of one of our frigates can afford. Our own conviction is, that this fever, as well as many others, confers a temporary immunity from second attacks, without proving the disease to be either contagious or specific. The question is involved in Cimmerian darkness—and the more we investigate it, the more we are convinced that we are far from possessing materials for a final adjustment of the litigation.

